# A Novel PAN/Apple Domain-Containing Protein from *Toxoplasma gondii*: Characterization and Receptor Identification

**DOI:** 10.1371/journal.pone.0030169

**Published:** 2012-01-19

**Authors:** Haiyan Gong, Kyousuke Kobayashi, Tatsuki Sugi, Hitoshi Takemae, Hitomi Kurokawa, Taisuke Horimoto, Hiroomi Akashi, Kentaro Kato

**Affiliations:** Department of Veterinary Microbiology, Graduate School of Agricultural and Life Sciences, University of Tokyo, Tokyo, Japan; University of Texas-Houston Medical School, United States of America

## Abstract

*Toxoplasma gondii* is an intracellular parasite that invades nucleated cells, causing toxoplasmosis in humans and animals worldwide. The extremely wide range of hosts susceptible to *T. gondii* is thought to be the result of interactions between *T. gondii* ligands and receptors on its target cells. In this study, a host cell-binding protein from *T. gondii* was characterized, and one of its receptors was identified. P104 (GenBank Access. No. CAJ20677) is 991 amino acids in length, containing a putative 26 amino acid signal peptide and 10 PAN/apple domains, and shows low homology to other identified PAN/apple domain-containing molecules. A 104-kDa host cell-binding protein was detected in the *T. gondii* lysate. Immunofluorescence assays detected P104 at the apical end of extracellular *T. gondii*. An Fc-fusion protein of the P104 N-terminus, which contains two PAN/apple domains, showed strong affinity for the mammalian and insect cells evaluated. This binding was not related to protein-protein or protein-lipid interactions, but to a protein-glycosaminoglycan (GAG) interaction. Chondroitin sulfate (CS), a kind of GAG, was shown to be involved in adhesion of the Fc-P104 N-terminus fusion protein to host cells. These results suggest that P104, expressed at the apical end of the extracellular parasite, may function as a ligand in the attachment of *T. gondii* to CS or other receptors on the host cell, facilitating invasion by the parasite.

## Introduction

*Toxoplasma gondii* is a common intracellular parasite that causes serious symptoms in immunocompromised individuals and pregnant women [1]. Following infection, the parasite can persist for the life of the organism; thus, approximately 50% of the world's population is currently carrying *T. gondii*. Extensive research into drug therapies for the treatment of toxoplasmosis has been carried out; however, most of the drugs in use are toxic [2], and *T. gondii* can readily develop drug resistance [3]. In fact, they are currently being administered to not only infected ordinary adults but also infected pregnant women and newborns who are more weak and susceptible to the toxicity [4].

The most promising measure for the protection of humans and animals against *T. gondii* infection is vaccination. Vaccination with SAG1, affinity-purified from the *T. gondii* RH strain, produced high survival rates and significantly decreased brain cyst loads in mice [5]–[8]. Also, the use of a combination of antigens delivered as plasmids coding for regions of micronemal proteins, including MIC2, MIC3, MIC4, M2AP, and AMA1, resulted in a significant reduction (84%) in the number of cysts [9]. Interestingly, almost all protective molecules seem to be involved in the parasite-host interaction [10]. Thus, the exploration of this type of molecule from *T. gondii* appears to be extremely important for vaccine development.

*Toxoplasma gondii* has the remarkable ability to invade a broad range of cell types. This parasite is believed to attach to host cells via ubiquitously expressed surface molecules of the host, or each host cell type may carry a unique receptor that is bound by a particular parasite molecule [11]. Fourteen PAN/apple domain proteins have been detected in *T. gondii* [12], although only two (TgAMA1 and TgMIC4) have been described [13], [14]. TgAMA1 was shown to form a complex, called a moving junction (MJ), with the neck of the rhoptries (for RON2/RON4/RON5 proteins) during the invasion process [15]. The depletion of TgAMA1 prevented MJ formation, and the parasite was consequently unable to invade host cells [16]. TgMIC4 has been shown to bind with and serve as a bridge between the parasite and host cell [13]. Since PAN/apple domain proteins from most species bind other proteins or carbohydrates [17], members of this family from *T. gondii* may mediate inter-specific interactions, thereby providing a link between host and parasite. To explore the function or characters of other members of the family, we selected a sequence containing several PAN/Apple domains from the GenBank, characterized the protein and identified one of its receptors on host cell surface.

Glycosaminoglycans (GAGs), or mucopolysaccharides, are long unbranched polysaccharides consisting of a repeating disaccharide unit [18]. GAGs include chondroitin sulfate (CS), dermatan sulfate, keratin sulfate, heparin, heparin sulfate (HS), and hyaluronan, among which CS is the most prevalent GAG component [19]. Cell surface GAGs are utilized as a receptor by a variety of pathogens, including *Chlamydia* [20], *Trypanosoma* [21], *Plasmodium* [22], and *Toxoplasma* [11], [18]. A surface antigen from *T. gondii*, SAG3, was shown to interact with HS on the surface of Chinese hamster ovary (CHO)-K1 cells [23]; however, other molecules that bind GAGs have not yet been identified. In the present study, CS was shown to bind a PAN/apple domain-containing protein from *T. gondii*, suggesting that various GAGs may function as receptors in parasite-host cell attachment.

## Materials and Methods

### Biochemical reagents and antibodies

CSA and CSC were purchased from Sigma-Aldrich. Rabbit anti-M2AP (microneme protein 2-associated protein) polyclonal antibodies were kindly provided by Dr. V. Carruthers (John Hopkins University, Baltimore, MD); rabbit anti-ROP1 polyclonal antibodies were a gift from Dr. J. Dubremetz (University of Montpellier, Montpellier, France); rabbit anti-GRA6 polyclonal antibodies were kindly sent to us by Dr. L.D. Sibley (Washington University School of Medicine, MO, USA)

### Parasites and cell culture

Tachyzoites of *T. gondii* RH strain [24] were inoculated in a monolayer of Vero cells [24] cultured in Dulbecco's modified essential medium (DMEM; Nissui, Tokyo, Japan) supplemented with 7.5% fetal bovine serum (FBS). 293T cells [24], [25] were cultured in DMEM with 10% FBS. CHO-K1 cells and two mutant strains of CHO-K1, *pgs*A-745 and *pgs*D-677 cells, were purchased from the American Type Culture Collection (ATCC) and cultured in F-12 (Gibco BRL, Grand Island, NY) medium containing 10% FBS. P3U1 [26], K562 (Riken BRC Cell Bank, Ibaraki, Japan), and Jurkat cells [27] were maintained in RPMI1640 medium (Sigma-Aldrich) with 10% FBS. Insect cells (*Spodoptera frugiperda* Sf9 [24], [25] and *Trichoplusia ni* Tn5 [25]) were cultured in Sf-900II SFM (Invitrogen, Carlsbad, CA) and Ex-cell 405 (SAFC Biosciences Inc., Lenexa, KS), respectively.

### Recombinant protein synthesis

**Figure 1:**
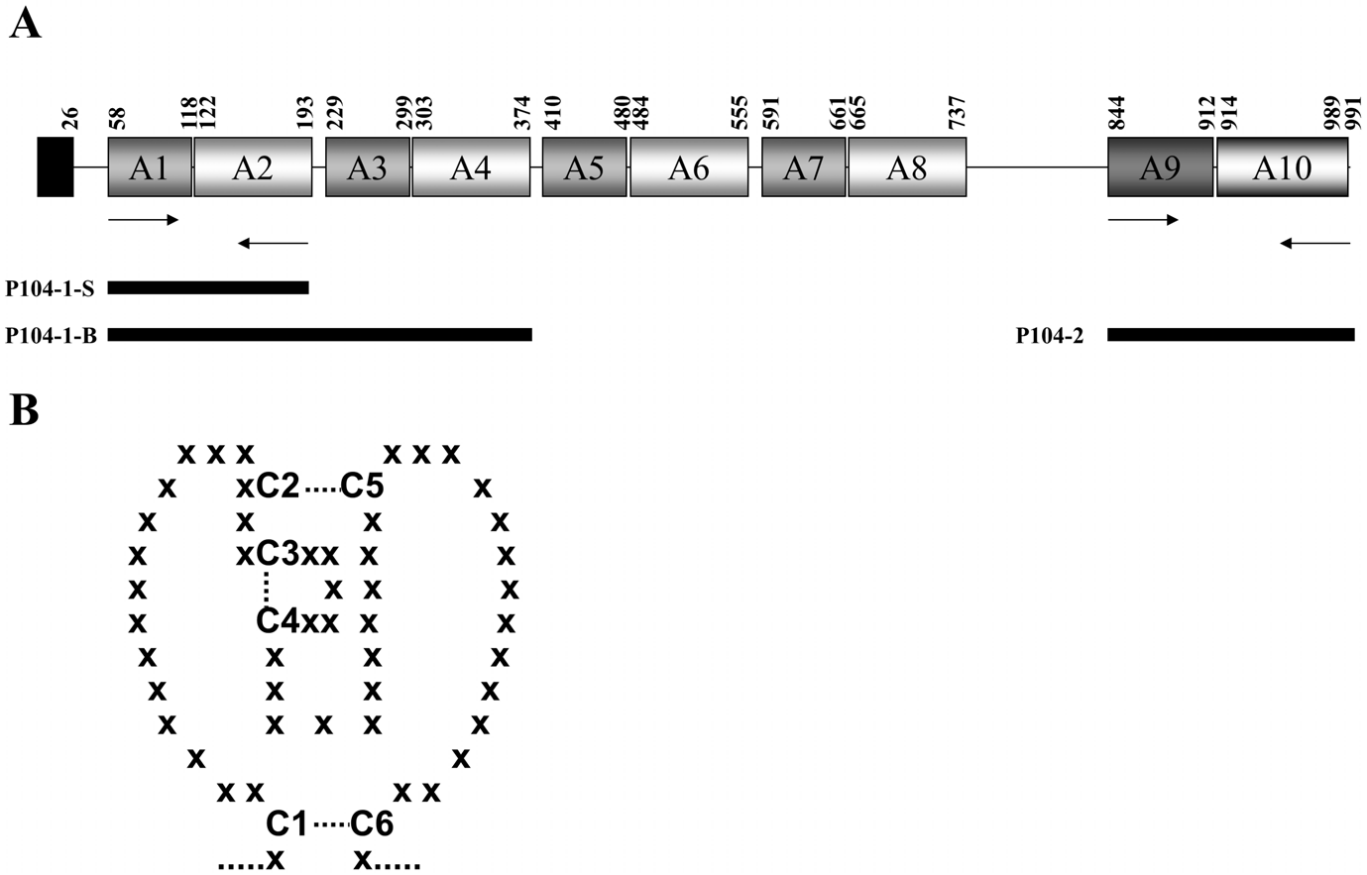
Analysis of the P104 protein sequence. A. The signal peptide is indicated by black rectangle. A1, A3, A5, and A7 are indicated by a dark gray rectangle. A2, A4, A6, and A8 are indicated by a light gray rectangle. A9 and A10 are shown in light black and shiny black rectangles. The arrows at the N- and C-termini indicate the designated primers; the corresponding PCR products are indicated by black bands. B. The putative apple-like structure of a PAN/apple domain. C1–C6 (shown in bold) indicate the six cysteine residues that form three disulfide bridges.

Using the sequence obtained from GenBank (CAJ20677), primers were designed for plasmid construction in pBSV-Fc-8His [25]. The N-terminus of the protein contains four repeats of similar amino acid residues; the forward primer, P104-1-Fc-F (5′-GGACTAGTAGAGGAAAGCCTGAATACAGTCAACG-3′; *Spe*I site underlined), and reverse primer, P104-1-Fc-R (5′-TGAATTCCACGATTCGGACTCCTCCTCAGT-3′; *Eco*RI site underlined), were designed to flank the ends of this repeat (Fig. 1A). The resulting PCR products contained one (P104-1-S) or two repeats (P104-1-B). The forward primer for the C-terminus was P104-2-Fc-F (5′-CAGGATCCGAGGCGCTGCCGGGTG-3′; *Bam*HI site underlined), while the reverse primer was P104-2-Fc-R (5′-TCACCCGGGGCAGAAATCCCTGGGACCGAC-3′; *Sma*I site underlined). The forward primer for the N-terminus of P104, constructed in pGEX-6P-2 and expressed in *E. coli*, was P104-1-GST-F (5′-GGAATTCCCAGAGGAAAGCCTGAATACAGTCAAC-3′; E*co*RI site underlined); the reverse primer was P104-1-GST-R (5′-GCGGCCGCCGATTCGGACTCCTCCTCAGT-3′; *Not*I site underlined). Construction of the plasmids and expression of the recombinant proteins in Tn5 insect cells was done as described previously [25] with the following modifications. Briefly, total RNA was prepared from *T. gondii* RH strain following propagation in Vero cells using Trizol reagent (Invitrogen). Next, RT-PCR was done using the SuperScript III one-step RT-PCR system with platinum Taq DNA polymerase (Invitrogen). The amplified products were cloned into pBSV-Fc-8His and their sequences confirmed. Subsequently, the positive clones were co-transfected with BaculoGold DNA (BD Biosciences, San Jose, CA) into Sf9 insect cells, and used to infect Tn5 cells. The fusion proteins, designated as rP104-1-S/Fc, rP104-1-B/Fc, and rP104-2/Fc, were purified from the lysate of the culture medium of the infected Tn5 cells. Moreover, the expression of Fc-recombinant proteins was confirmed by Western blotting using anti-mouse Fc antibody. Expression of the GST-recombinant protein (rP104-1/GST) in pGEX-6P-2 was carried out according to the manufacturer's protocol.

### Anti-rP104-1/GST serum preparation, Western blotting, and immunofluorescence assay (IFA)

Mice were immunized three times with rP104-1/GST to produce anti-rP104-1/GST antibodies. This work was approved by the Research Ethics Review Committee of Graduate School of Agricultural and Life Sciences, the University of Tokyo (Approval no. P08-183). Anti-sera and normal sera were prepared and collected as described previously [28]. For Western blotting, strain RH was propagated and lysed by passing the cells though a #27 syringe and filtered using a 5-µm filter. The purified tachyzoites were lysed in 1× SDS-PAGE buffer, boiled for 5 min, and subjected to 10% SDS-PAGE and Western blotting using Immun-Blot PVDF membranes (Bio-Rad). The primary antibody was diluted 1∶100 in phosphate-buffered saline [PBS] with 0.1% Tween 20 and 1% BSA and incubated with the membrane for 1 h. Detection was performed as described previously [28]. For IFA, purified tachyzoites were fixed on a 14-well slide and permeabilized with 0.1% Triton X-100. The slide was then reacted with anti-rP104-1-S/GST antibodies followed by goat anti-mouse IgG-Alexa Fluor 488 (Invitrogen). Rabbit anti-M2AP, anti-ROP1 and anti-GRA6 antibodies were employed for co-localization assays with P104 in *T. gondii*. Intracellular *T. gondii* was prepared by the infection of Vero cells cultured in an eight-well chamber slide for 48 h; the slide was fixed and stained as described above. Initial attempts to localize P104 in intracellular *T. gondii* relied on antibodies to rP104-1-S/GST were unsuccessful. As an alternative strategy, we introduced a FLAG tag to the C-terminus of P104 and transfected into RH parasites using pMini.3×Flag.ht. vector under the control of GRA1 promoter [29]. Firstly, genome DNA was extracted from purified *T. gondii* and subjected to a PCR reaction using the following primers, which generated restriction sites *Eco*T22I and *Bgl*II: 5′-AGAAATCAAGCAAGATGTGGAAGTACGGATTTTTTCTGACAG-3′ (sense) and 5′CTGGTACCGATATCAGAGAAGGGCAGAAATCCCTGGGAC-3′ (antisense). The purified PCR product was blunted with DNA blunting kit (TaKaRa, Japan) and then inserted into digested and blunted pMini.3×Flag.ht. vector using In-Fusion HD EcoDry cloning kit (Clontech, Japan). The 15 bp overlap was underlined in the primers. Fifty microgram plasmid of identified positive clone was transiently transfected into 1×10^6^ purified *T. gondii* according to the description elsewhere [30]. And the transfected parasites were inoculated into Vero cells cultured in 8-well slide. After 36 h of infection, the parasites were double stained with mouse anti-FLAG and rabbit anti-M2AP, anti-ROP1 or anti-GRA6 antibodies, respectively.

### Flow cytometry

Several types of erythrocytes (1×10^5^ cells) were incubated with 1.5 pmol of rP104-1-S/Fc, rP104-1-B/Fc, or rP104-2/Fc in fluorescence activated cell sorting (FACS) buffer (PBS with 2% FCS and 0.1% NaN_3_) for 1 h at 4°C. Binding of the recombinant proteins was measured as described previously [25]. For protease treatment, Vero, K562, and P3U1 cells were treated with 0.1 mg/ml trypsin or chymotrypsin for 2 h at room temperature. Next, 1 mg/ml protease inhibitor was added to stop the reaction. After washing with PBS three times, the cells were subjected to the same incubation with 1.5 pmol of rP104-1-S/Fc followed by FACS analysis, as described above. Flow cytometric analysis was done using WinMDI version 2.9 software.

### Lipid binding assay

To investigate the lipid binding activity of rP104-1-S/Fc, a protein-lipid overlay assay was carried out using Membrane Lipid Strips (Echelon Biosciences, Salt Lake City, UT) according to the manufacturer's protocol. The strip is a hydrophobic membrane pre-spotted with 15 different major biologically active lipids found in cell membranes (100 pmol per spot). It was blocked with 1% gelatin protein (MoBiTech, Goettingen, Germany) and 1% Tween 20 (Wako, Tokyo, Japan) in PBS (PBS-T) at room temperature for 1 h, then incubated with 5 nM rP104-1-S/Fc at room temperature for 1 h with gentle shaking. Fc protein expressed from pBSV-Fc-8His was used as a negative control. After three washes, anti-mouse-HRP conjugate (1∶5,000) in PBS with 1% gelatin protein was added and the signal was detected by enhanced chemiluminescence (ECL; Amersham Biosciences UK Ltd., Buckinghamshire, UK). All reactions were performed in the dark.

### Inhibition of rP104-1-S/Fc binding activity by CSA and CSC

Different concentrations of CSA and CSC (0.1–20 mg/ml) were prepared in PBS and incubated with 1.5 pmol of rP104-1-S/Fc for 1 h at 4°C, after which the mixture was incubated with CHO-K1 cells (1×10^5^ cells) for 1 h at 4°C. The binding activity was analyzed by flow cytometry as described above. To confirm the binding of rP104-1-S/Fc to CS on the host cells, two mutant CHO-K1 cell lines, *pgs*A-745 (xylosyltransferase deficient, causing defects in HS and CS) [31] and *pgs*D-677 (lacks acetylglucosaminyltransferase and glucuronyltransferase activity, causing depletion of HS and increasing the concentration of CS three folds) [11], were subjected to a binding assay with 1.5 pmol of rP104-1-S/Fc and FACS analysis as described above. All experiments were done in triplicate.

### CSA-affinity chromatography

One milligram of CSA was coupled to cyanogen bromide (CNBr)-activated Sepharose (Sigma-Aldrich) according to the manufacturer's protocol. The CSA-conjugated beads were incubated with rP104-1-S/Fc in NP-40 lysis buffer (50 mM Tris-HCl, 150 mM NaCl, and 1% NP-40, pH 8.0) overnight at 4°C. The beads were then washed extensively with lysis buffer four times and eluted in 1× SDS-PAGE sample buffer. The eluate was subjected to 10% SDS-PAGE and transferred to a PVDF membrane (Bio-Rad). After blocking with 1% BSA in PBS-T, the signal was detected using HRP-conjugated anti-mouse Fc antibodies. Beads treated with coupling buffer instead of CSA were employed as a mock trial.

### *In vitro* inhibition of *T. gondii* invasion by rP104-1-S/Fc

A total of 1×10^4^ Vero cells were seeded in an 8-well chamber and incubated for 1 h with 0.6 or 1.5 µM rP104-1-S/Fc or Fc diluted in infection medium (2% FBS in DMEM). The cells were then inoculated with 2×10^5^ particles of *T. gondii*-expressing green fluorescent protein (GFP). Two hours after infection, the chamber was gently washed with PBS three times before the addition of mouse anti-SAG1 antibodies (TP3 diluted 1∶1,000 in PBS; HyTest Ltd., Turku, Finland). After 30 min of staining for SAG1 on the extracellular parasite, the chamber was washed with PBS three times, fixed, permeabilized, and incubated with goat anti-mouse secondary antibodies labeled with Alexa Fluor 546 (Invitrogen). Simultaneously, TO-PRO-3 was used to stain the nuclei of the host cells. Finally, the slide was observed and images collected with an LSM510 confocal microscope (Zeiss). The invasion rate was calculated as the ratio of parasites per cell in the test group compared to that in the control group (without added protein). To investigate the effect of CSA on parasite invasion, various concentrations of CSA (0–2.5 mg/ml) were added to Vero cells prior to infection with *T. gondii*.

### Statistical analysis

Student's *t* test was used to determine the statistical significance.

## Results

### Sequencing of P104 and the recombinant proteins

**Figure 2:**
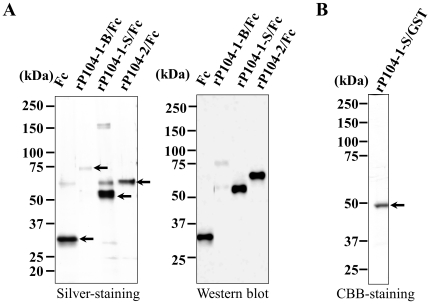
Recombinant proteins synthesized by eukaryotic and prokaryotic expression systems. A. Left panel, silver-stained gel showing the recombinant proteins with their expected molecular weights purified from the Tn5 (insect) cell culture medium. Right panel, purified recombinant proteins were transferred onto a PVDF membrane and anti-mouse Fc antibody was employed to detect the Fc-recombinant protein bands. B. CBB-stained gel showing the fusion protein rP104-1-S/GST purified from *E. coli*. The purified proteins are shown by arrows. The molecular masses in kDa are shown on the left.

The primary sequence of P104 is composed of 10 putative PAN/apple domains, according to an analysis done using Prosite (www.expasy.ch/cgi-bin/prosite). Each domain contains six cysteine residues that form three disulfide bonds, creating an apple-like structure (Fig. 1B). The second to fifth cysteine residues match the consensus sequence seen in TgMIC4 (CX_3_CX_5_CX_11_C) [13]. These 10 PAN/apple domains are arranged as follows: A1 (residues 58–118), A2 (122–193), A3 (229–299), A4 (303–374), A5 (410–480), A6 (484–555), A7 (591–661), A8 (665–737), A9 (844–912), and A10 (914–989). Interestingly, A1 and A2 were repeated successively four times in the primary sequence (Fig. 1A). Here, A1 and A2 were amplified as P104-1-S, while A1–A4 were amplified as P104-1-B; A9 and A10 were amplified as P104-2. The P104 protein sequence showed low homology to other identified PAN/apple domain-containing proteins, but approximately 80% homology to m01899 (ToxoDB.org v4.1), as shown in the supplemental data of a previous study [12]. However, m01899 has not been characterized to date. For further analysis, rP104-1-S/Fc, rP104-1-B/Fc and rP104-2/Fc were synthesized and confirmed by Western blot analysis (Fig. 2A). To prepare anti-serum, rP104-1-S/GST was expressed and purified (Fig. 2B) for immunization of mice.

### Identification of P104 in *T. gondii*

**Figure 3:**
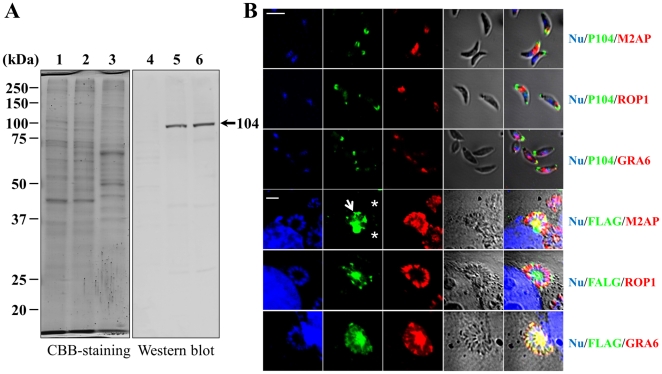
Localization of P104 in intracellular and extracellular *T. gondii*. A. Western blot analysis of P104 in the *T. gondii* lysate. Lane 1 and 4, Vero cell lysate; lane 2 and 5, lysate of *T. gondii*-infected Vero cells; lane 3 and 6, lysate of purified *T. gondii*. Lane 1–3, CBB-staining of Vero cell and *T. gondii* lysate; lane 4–6, Western blotting of the lysates. Mouse anti-rP104-1-S/GST was used as the primary antibody. The molecular masses in kDa are shown on the left. B. Co-localization assays of P104 with other proteins in extracellular (upper 3 panels) and intracellular (lower 3 panels) *T. gondii*. Green, anti-rP104-1-S/GST antibodies; red, anti-M2AP, anti-ROP1 and anti-GRA6 antibodies; blue, nuclei (with TO-PRO-3 staining). In intracellular *T. gondii* (lower 3 panels), mouse anti-FLAG antibody, instead of anti-rP104-1-S/GST, was used to stain the recombinant protein of P104 with a C-terminal FLAG tag (green stained, arrows). Wild-type parasites that failed to be stained with anti-FLAG antibody were indicated with stars.

Anti-rP104-1-S/GST antibodies were prepared in mice to enable the detection of P104 in *T. gondii*. As expected, a band with a molecular size of approximately 104 kDa was found in the lysate of purified *T. gondii* and Vero cells infected with *T. gondii*, while no band appeared in the uninfected Vero cell lysate (Fig. 3A). The co-localization assays of P104 with M2AP, ROP1 and GRA6 indicates that P104 was not expressed in the microneme, rhoptry or dense granules, but at the apical end of extracellular *T. gondii*, (Fig. 3B, upper panels). Because of unknown reason, we failed to specifically localize P104 protein in intracellular parasites using our mouse antibodies against rP104-1-S/GST. Therefore, The ORF of *P104* gene was tagged with FLAG and transfected into RH parasites. As shown in Fig. 3B (lower panels), detection with anti-FLAG antibody revealed that P104 protein was expressed and secreted into parasitophorous vacuole (PV) and co-localized with GRA6 in transfected intracellular parasite (shown by arrows), while wild-type parasites did not stain with anti-FLAG antibody (shown by stars).

### Binding of the Fc-recombinant proteins to cells

**Figure 4:**
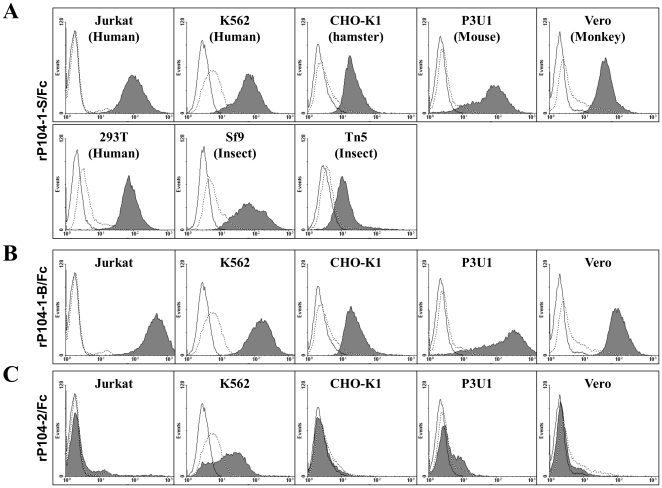
FACS analysis of the cellular binding activity of rP104-1-S/Fc and rP104-1-B/Fc. A. Different types of cells were incubated with rP104-1-S/Fc before being stained with anti-Fc-FITC antibodies and subjected to FACS analysis with Cell Quest software. B. FACS analysis of cells incubated with rP104-1-B/Fc. C. FACS analysis of cells incubated with rP104-2/Fc. In all panels, cells (solid line) and cells incubated with Fc (dotted line) were employed as negative controls. Gray-filled histograms indicate the cells incubated with recombinant proteins.

rP104-1-S/Fc and rP104-1-B/Fc were able to attach to all of the cell types examined, including Jurkat, K562, CHO-K1, P3U1, and Vero cells (Fig. 4A and B); in contrast, rP104-2/Fc did not show specific affinity for any of the tested cells (Fig. 4C). Besides mammalian cells, rP104-1-S/Fc was shown to bind with two types of insect cells, Sf9 and Tn5 cells (Fig. 4A).

### The role of cell surface proteins or lipids in binding to rP104-1-S/Fc

**Figure 5:**
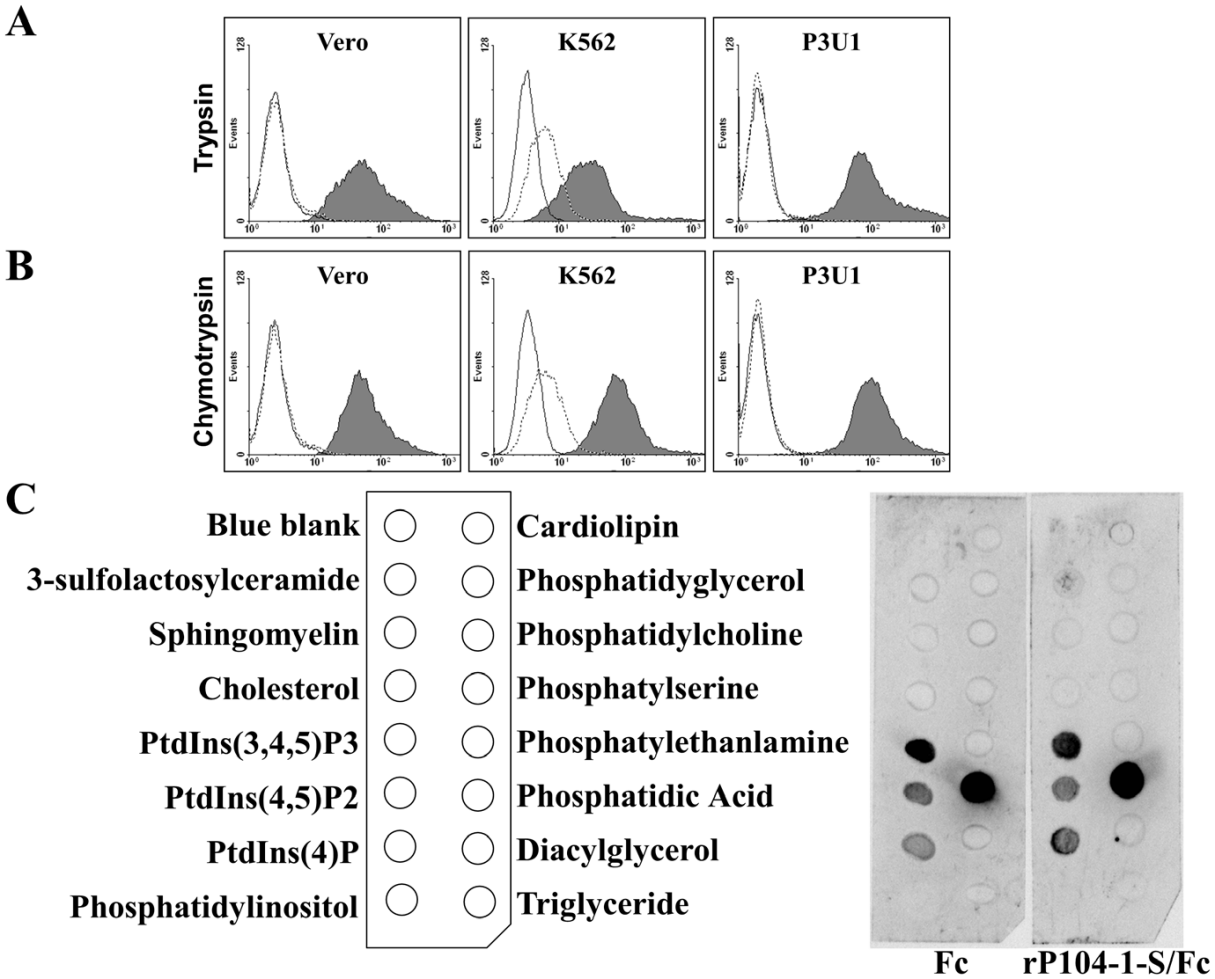
The attachment of rP104-1-S/Fc to host cells was not affected by protease digestion of cell surface proteins, and the receptor was not a major lipid on the cell surface. Vero, K562, and P3U1 cells were treated with trypsin (A) or chymotrypsin (B) to remove surface proteins, and then incubated with rP104-1-S/Fc (gray-filled histograms) or Fc (dotted line). The bound signals were detected by FACS analysis. The solid line indicates the cell-only control. C. rP104-1-S/Fc was incubated with a membrane spotted with 15 major lipids from the cell surface (right panel). The membrane was then immersed in HRP-conjugated anti-mouse antibodies, and reaction spots were revealed using the ECL system. Fc was used as a negative control (central panel). The names of lipids were shown on the left panel.

To investigate the receptor for rP104-1-S/Fc, trypsin and chymotrypsin were used to digest the surface proteins of Vero, K562, and P3U1 cells. Trypsin favors basic residues such as lysine and arginine, while chymotrypsin favors aromatic residues such as phenylalanine, tyrosine, and tryptophan [32]. On hepatoma tissue culture (HTC) cells, almost all surface proteins are sensitive, to some degree, to proteolysis by trypsin (with the exclusion of some glycoproteins) [33]. To confirm surface protein disruption by the proteases, the treated cells were incubated with a second protein known to interact with protein on the untreated cells, and attachment was completely ablated (unpublished data). This suggests that the proteins on the cell surface were effectively destroyed, while rP104-1-S/Fc adhesion to the host cells was unaffected (Fig. 5A and B). Thus, rP104-1-S/Fc likely does not adhere to cells through protein receptors that are sensitive to digestion by trypsin or chymotrypsin. In a subsequent experiment, a nitrocellulose membrane lipid strip spotted with 15 different biologically abundant lipids found in cell membranes was interacted with rP104-1-S/Fc or Fc. Interestingly, rP104-1-S/Fc showed the same reaction as the negative control (Fc). Both proteins reacted with phosphatidic acid (PA), phosphatidylinositol (PtdIns)(3,4,5)P3, PtdIns(4,5)P2, and PtdIns(4) (Fig. 5C), which suggests that rP014-1-S/Fc bound to the above lipids through the Fc fragment but not P104-1-S portion. Therefore, none of the examined 15 types of lipids functions as a receptor for P104-1-S. The reason for the binding of Fc to lipids is unknown yet.

### Attachment of rP104-1-S/Fc to CS

**Figure 6:**
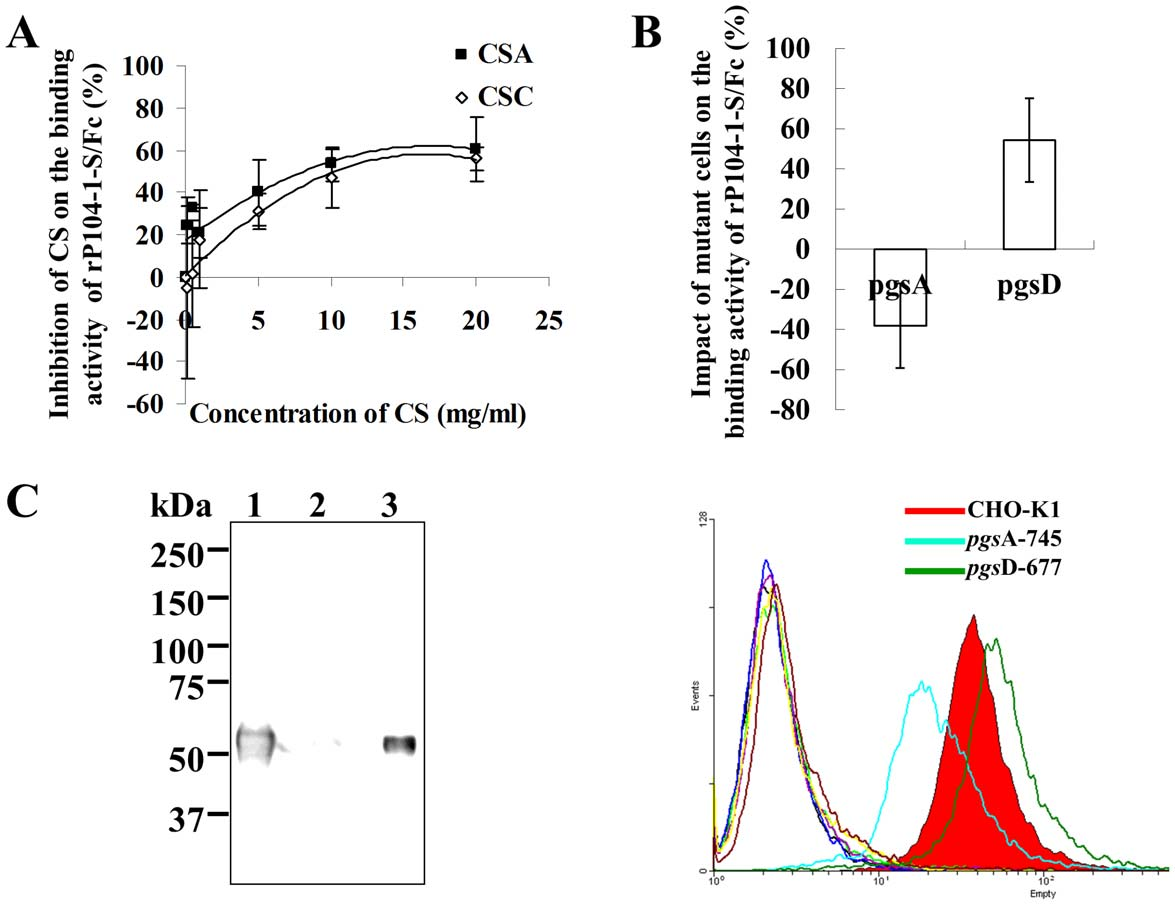
CS is necessary for the adhesion of rP104-1-S/Fc to cells. A. Inhibitory effect of CS on the binding activity of rP104-1-S/Fc to CHO-K1 cells. The inhibitory effect of CSA and CSC on the activity of rP104-1-S/Fc was calculated as the ratio of the geometric mean fluorescence intensity value (GMean): (1-CSA-treated cells/no treated cells) ×100. The experiment was performed in triplicate. B. Binding activity of rP104-1-S/Fc to CHO-K1 mutant cells. The upper panel shows the ratio of the GMean as (rP104-1-S/Fc-mutant cells/rP104-1-S/Fc-wild cells-1) ×100; the experiment was performed in triplicate. The lower panel shows the change in fluorescence as evaluated by FACS assay. Different colors indicate the different types of cells and treatments: CHO-K1 (black), Fc-treated CHO-K1 (light green), rP104-1-S/Fc-treated CHO-K1 (red filled histogram), *pgs*A-745 (dark blue), Fc-treated *pgs*A-745 (purple), rP104-1-S/Fc-treated *pgs*A-745 (light blue), *pgs*D-677 (yellow), Fc-treated *pgs*D-677 (dark red), and rP104-1-S/Fc-treated *pgs*D-677 (dark green). C. Binding of CSA-conjugated beads to rP104-1-S/Fc. CSA-conjugated beads were incubated with rP104-1-S/Fc and then stained with HRP-conjugated anti-mouse antibodies. The bands indicate that rP104-1-S/Fc was co-purified with the CSA-coupled beads. Lane 1, input rP104-1-S/Fc; 2, mock beads incubated with rP104-1-S/Fc; 3, CSA-treated beads incubated with rP104-1-S/Fc. The molecular masses in kDa are shown on the left.

rP104-1-S/Fc was incubated with various glycans, including glucose, lactose, CSA, and CSC, for 1 h at 4°C in a binding assay with 293T, CHO-K1, or Vero cells. CSA and CSC significantly inhibited the binding activity of rP104-1-S/Fc (data not shown). Moreover, CSA and CSC inhibited the binding of rP104-1-S/Fc to CHO-K1 cells in a dose-dependent manner. When the concentration of CSA or CSC was greater than 1 mg/ml, CSA showed slightly higher inhibitory activity toward rP104-1-S/Fc than CSC (Fig. 6A). To corroborate this binding model, two mutants of CHO-K1 cells, *pgs*A-745 and *pgs*D-677, were used in the binding assay, and our results demonstrate that *pgs*A-745 decreased the binding trend while *pgs*D-677 increased this trend by 54% when the binding activity of rP104-1-S/Fc to wild type of CHO-K1 was considered as zero (Fig. 6B). Furthermore, CSA was coupled to CNBr-activated beads and incubated with rP104-1-S/Fc. rP104-1-S/Fc was co-purified with the CSA-conjugated beads, but not with control beads (Fig. 6C).

### *In vitro* inhibitory effect of rP104-1-S/Fc on *T. gondii* invasion

**Figure 7:**
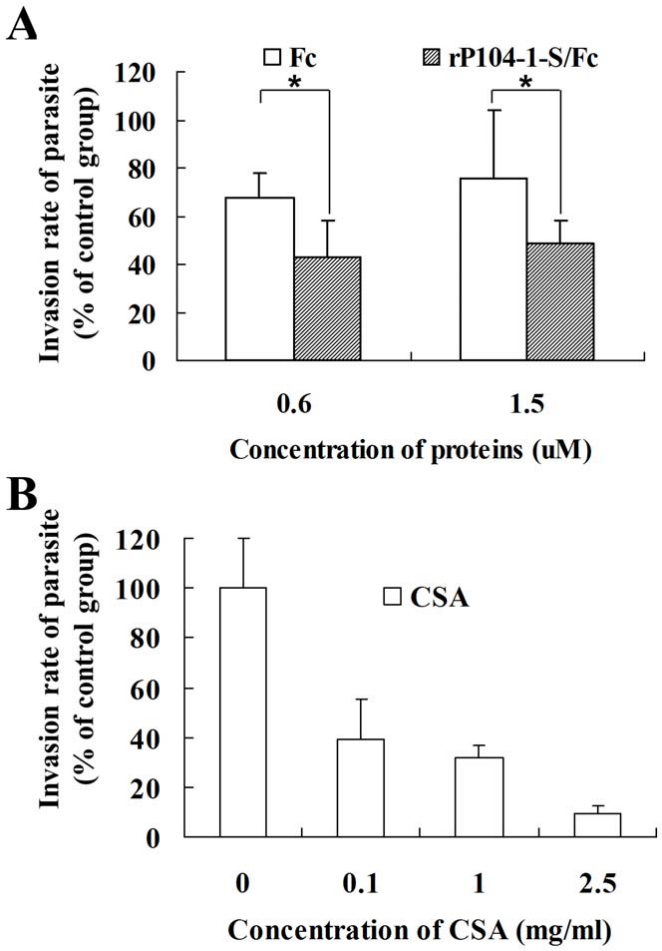
*In vitro* inhibitory effect of rP104-1-S/Fc or CSA on the invasion of *T. gondii*. Monolayers of Vero cells in an 8-well chamber slide were infected with 2×10^5^ GFP-expressing *T. gondii* after incubation with different concentrations of rP104-1-S/Fc (A) or CSA (B). Extracellular and intracellular parasites were stained differentially and enumerated as described in method section. The invasion rate was calculated as the ratio of parasites/cells in the tested group compared to that in the mock group (Vero cells treated with no protein prior to infection with *T. gondii*). Fc was used as a negative control. Student's *t*-test, asterisk, P<0.05; mean ± standard deviation (n = 3 experiments).

To evaluate the significance of rP104-1-S/Fc as a vaccine candidate, we added rP104-1-S/Fc to Vero cells to block its receptors, and then investigated the invasion rate of *T. gondii*. With the invasion rate of the control group (no protein added) set at 100%, treatment with Fc and rP104-1-S/Fc decreased the infection rate. Compared with the Fc-treated group, rP104-1-S/Fc significantly affected the infection rate of the parasite on the concentrations of both 0.6 µM (67.75±10.06 versus 42.97±15.37, student' s *t* test, P<0.05) and 1.5 µM (75.28±20.59 versus 48.60±9.86, student' s *t* test, P<0.05) (Fig. 7A). As a receptor for rP104-1-S/Fc, exogenous CSA may compete with CSA on the cell surface for adherence to *T. gondii*, thus impeding invasion of the parasite. As expected, the addition of CSA inhibited the invasion of *T. gondii* in a dose-dependent manner (Fig. 7B).

## Discussion

The apple domain is a subset of PAN superfamily, which is widely detected in various organisms, including bacteria [34], apicomplexcans [13], [35]–[39], filamentous fungi [40], plants [41], nematodes [17], amphibians [42], avians [43], and mammals [44]. These molecules all contain a PAN/apple domain that mediates protein-protein or protein-carbohydrate interactions. As mentioned, TgAMA1 [14] and TgMIC4 [13] contain 2 and 6 PAN/apple domains, respectively, which play an important role in the invasion process [16], [45]. This study identified a third PAN/apple domain-containing protein, P104, which contains 10 putative PAN/apple domains. This large number of PAN/apple domains may function as well as those found in *Eimeria tenella* MIC5 (EtMIC5) [35], which facilitates the molecules to project away from the parasite surface [46]. This is consistent with the role of EtMIC5 in host cell binding [47]. Double staining of extracellular *T. gondii* revealed an unexpected result that P104 was not co-localized with proteins from the microneme, rhoptry or dense granules, but appeared on the apical end and possible conoid of the parasite (Fig. 3B). It is a coincidence that Morrissette *et al.* have previously detected a protein of 104 kDa at the apical part of *T. gondii*, with exclusion of microneme, rhoptry or dense granules, using monoclonal antibodies which specifically recognize the extreme apex of the parasite [48]. Although the intracellular expression of P104 (as a recombinant protein with FLAG tag) in PV, as observed to co-localize with GRA6, is not consistent with the one that Morrissette *et al.* have described, which may be attributed to the effect of the GRA1 promoter that possibly misguided the secretion of the recombinant protein in transfected parasite, it is intriguing to determine whether P104 in this study is the same one as mentioned by Morrissette *et al.* Since we did not specifically detect the expression of P104 in intracellular parasite using mouse anti-rP104-1-S/GST antibody, other experiment is necessary for the confirmation of the expression model of P104.

In this study, rP104-1-S/Fc bound various types of mammalian and two types of insect cells, which may hint that *T. gondii* has evolved some proteins to adhere the ubiquitously expressing molecules thus extended its host range [49]. The recombinant protein of A9 and A10 domains in P104 (rP104-2/Fc) showed low binding activity to K562 cells (Fig. 4C). This attachment may be mediated by an interaction of the Fc fragment on the fusion protein with the 40-kDa Fc receptor (Fc gamma RII) on K562 cells [50], though the function of A9 and A10 remains unknown.

Previous work suggests that *T. gondii* uses sulfated proteoglycans as substrates for host cell attachment. Exogenous CSA and CSC significantly affected the gliding motility and disrupted the adhesion of *T. gondii* to human fibroblasts at high concentrations [18]. Removal of chondroitin sulphate A, B and C on cell surface decreased *Neospora caninum* binding to Vero cells [51]. CS is also necessary for invasion of the mosquito midgut by *Plasmodium* ookinetes [52], which suggests an important role for CSA in the invasion of apicomplexans. In *N. caninum*, NcMIC4 [36] and NcMIC3 [51] were proved ligands for chondroitin sulphate binding. In *Plasmodium*, Duffy binding-like domains possess CSA binding activity and can protect against pregnancy-associated malaria [53]. However, the ligands of CS in *Toxoplasma* remain unknown. This study revealed the protein employed by *T. gondii* to attach to CS on host cells. Cells defective in CS showed reduced binding activity to rP104-1-S/Fc, and rP104-1-S/Fc was co-purified with CSA-coupled beads (Fig. 6). This confirmed the interaction of rP104-1-S with CS. However, CSA and CSC did not completely inhibit the binding of rP104-1-S/Fc to host cells in our study. Even mutant cells depleted of GAGs were not entirely resistant to rP104-1-S/Fc binding, suggesting that other molecules are also involved in the interaction. This hypothesis requires further investigation.

In this study, the addition of rP104-1-S/Fc significantly decreased the invasion rate of *T. gondii*, which suggests that exogenous rP104-1-S/Fc competitively adheres to CSA, CSC, or some other molecule, and partially affects host cell attachment and invasion by the parasite. Though the protection of rP104-1-S/Fc was not as prominent as that of SAG1, GRA1, GRA4 or GRA7 [54], it may be developed to co-operate with other proteins to vaccinate against *Toxoplasma*. CSA seriously impacted the infectivity of the parasite, consistent with previous results [18]. The results indicate that the control protein Fc decreased invasion by *T. gondii*. This may be explained by Fc receptor activity on the *T. gondii* tachyzoite. In this study, the Fc fragment was derived from mouse IgG2a. A decade ago, mouse monoclonal IgG2a antibodies were shown to bind the *T. gondii* membrane, and it was found that the binding site was not the Fab domain but the Fc fragment [55].

PAN/apple domain proteins in *T. gondii* may perform different, but important, functions separately or collaboratively. Therefore, our work is crucial for understanding the molecules that are involved in the invasion process, and could lead to the development of drugs or vaccine candidates for *T. gondii*.
